# Study of surface modification strategies to create glassy carbon-supported, aptamer-based sensors for continuous molecular monitoring

**DOI:** 10.1007/s00216-022-04015-5

**Published:** 2022-03-30

**Authors:** Miguel Aller Pellitero, Netzahualcóyotl Arroyo-Currás

**Affiliations:** 1 Department of Pharmacology and Molecular Sciences, Johns Hopkins University School of Medicine, Hunterian Building, Room 314, 725 North Wolfe St., Baltimore, MD 21205, USA; 2 Department of Chemical and Biomolecular Engineering, Johns Hopkins University, Baltimore, MD 21218, USA

**Keywords:** Diazonium salts, Amine grafting, Electrooxidation, Carbon biosensors, Continuous biosensing, Aptamer-based sensing

## Abstract

Electrochemical, aptamer-based (E-AB) sensors uniquely enable reagentless, reversible, and continuous molecular monitoring in biological fluids. Because of this ability, E-AB sensors have been proposed for therapeutic drug monitoring. However, to achieve translation from the bench to the clinic, E-AB sensors should ideally operate reliably and continuously for periods of days. Instead, because these sensors are typically fabricated on gold surfaces via self-assembly of alkanethiols that are prone to desorption from electrode surfaces, they undergo significant signal losses in just hours. To overcome this problem, our group is attempting to migrate E-AB sensor interfaces away from thiol-on-gold assembly towards stronger covalent bonds. Here, we explore the modification of carbon electrodes as an alternative substrate for E-AB sensors. We investigated three strategies to functionalize carbon surfaces: (I) anodization to generate surface carboxylic groups, (II) electrografting of arenediazonium ions, and (III) electrografting of primary aliphatic amines. Our results indicate that electrografting of primary aliphatic amines is the only strategy achieving monolayer organization and packing densities closely comparable to those obtained by alkanethiols on gold. In addition, the resulting monolayers enable covalent tethering of DNA aptamers and support electrochemical sensing of small molecule targets or complimentary DNA strands. These monolayers also achieve superior stability under continuous voltammetric interrogation in biological fluids relative to benchmark thiol-on-gold monolayers when a positive voltage scan window is used. Based on these results, we postulate the electrografting of primary aliphatic amines as a path forward to develop carbon-supported E-AB sensors with increased operational stability.

## Introduction

Electrochemical biosensors are powerful analytical tools that combine the specificity of biorecognition elements with the sensitivity of electrochemical detection [1]. These sensors mimic biology by specifically transducing molecular recognition events into electrical readouts that are proportional to the concentration of analyte. In addition, electrochemical interrogation can enable both single-point and continuous measurements, irrespective of sensor architecture. Coupled to these properties are advances in microfabrication and the development of low-cost, low-power portable electronics that offer the versatility to deploy electrochemical biosensors in an array of applications including food safety [2], environmental control [3], and health monitoring [4]. Although different recognition elements are available for the fabrication of such biosensors (e.g., enzymes, antibodies, and DNA/RNA), nucleic acid aptamers are uniquely suited to the task of real-time, continuous molecular monitoring. This is because, unlike enzymes or antibodies, aptamers can be rapidly and reproducibly developed in vitro, and offer the ability to reversibly bind virtually any biomolecular target irrespective of its reactivity [5]. Moreover, the rational design of aptamer sequences also offers the possibility to build receptors that undergo binding-induced conformation switching, which uniquely enables reversible molecular recognition in complex media such as in biological fluids [6]. Thus, electrochemical, aptamer-based (E-AB) sensors are especially attractive for continuous monitoring of biologically relevant molecules in vivo [7].

**Fig. 1:**
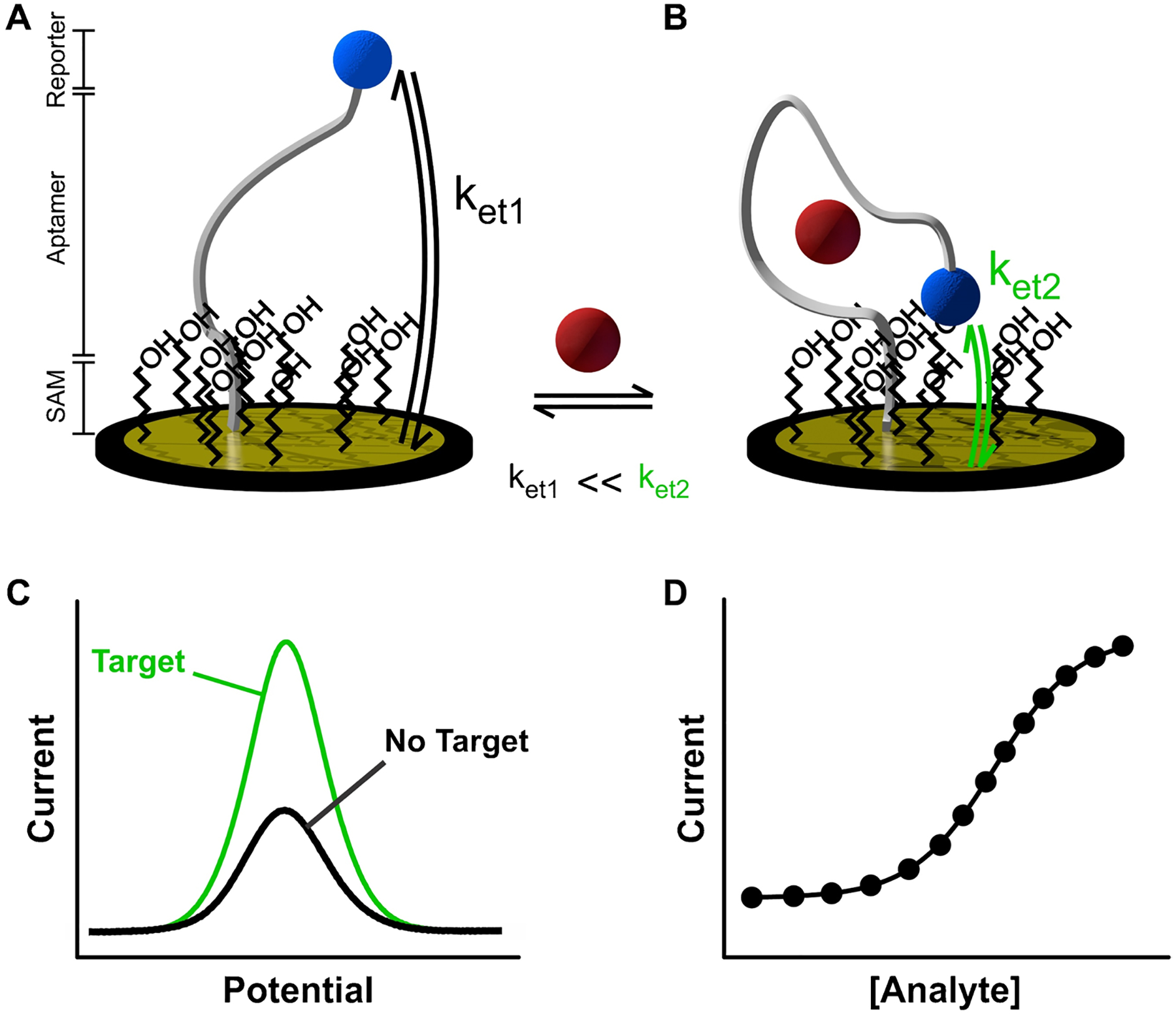
Architecture of E-AB sensors. **A** E-AB sensors generally comprise three elements: a blocking monolayer of short chain alkanethiols (SAM), target-binding nucleic acid aptamers, and a redox reporter (e.g., methylene blue) attached to the terminal end of the aptamer. **B** In the presence of target, aptamers undergo reversible binding-induced changes in conformation that alter the electron transfer rate (*k*_et_) between the redox reporter and the electrode surface, with *k*_et1_ < < *k*_et2_. **C** These changes in electron transfer can be monitored via changes in electrochemical currents, usually done via square wave voltammetry. **D** The relative change in peak currents can be monotonically correlated with target concentration in the sample via calibration curves that obey thermodynamic binding equilibria

Benchmark E-AB sensors consist of mixed monolayers containing blocking aliphatic and aptamer-modified thiols self-assembled onto gold surfaces (Fig. 1A). Aptamers are covalently labeled with a redox reporter, typically methylene blue, which can transfer electrons to the underlying gold surface. In the presence of target, aptamers undergo reversible, binding-induced changes in electron transfer (Fig. 1B), which can be monitored electrochemically (Fig. 1C). This signaling mechanism can be fast relative to biological metabolic processes, in the order of milliseconds [8], thus allowing for rapid equilibration of the aptamers with target levels (Fig. 1D) and enabling real-time molecular monitoring. Unfortunately, despite their successful implementation in continuous molecular monitoring in vivo [5, 7, 9–15], the short lifetime of E-AB sensors under continuous voltage cycling [16] — normally less than 12 h when deployed in vivo — precludes their use for health monitoring applications in humans, where relevant physiological processes occur in time scales of days or weeks. Thus, current efforts in the field are focused on developing strategies to significantly extend the operational life of E-AB sensors in biofluids.

**Fig. 2:**
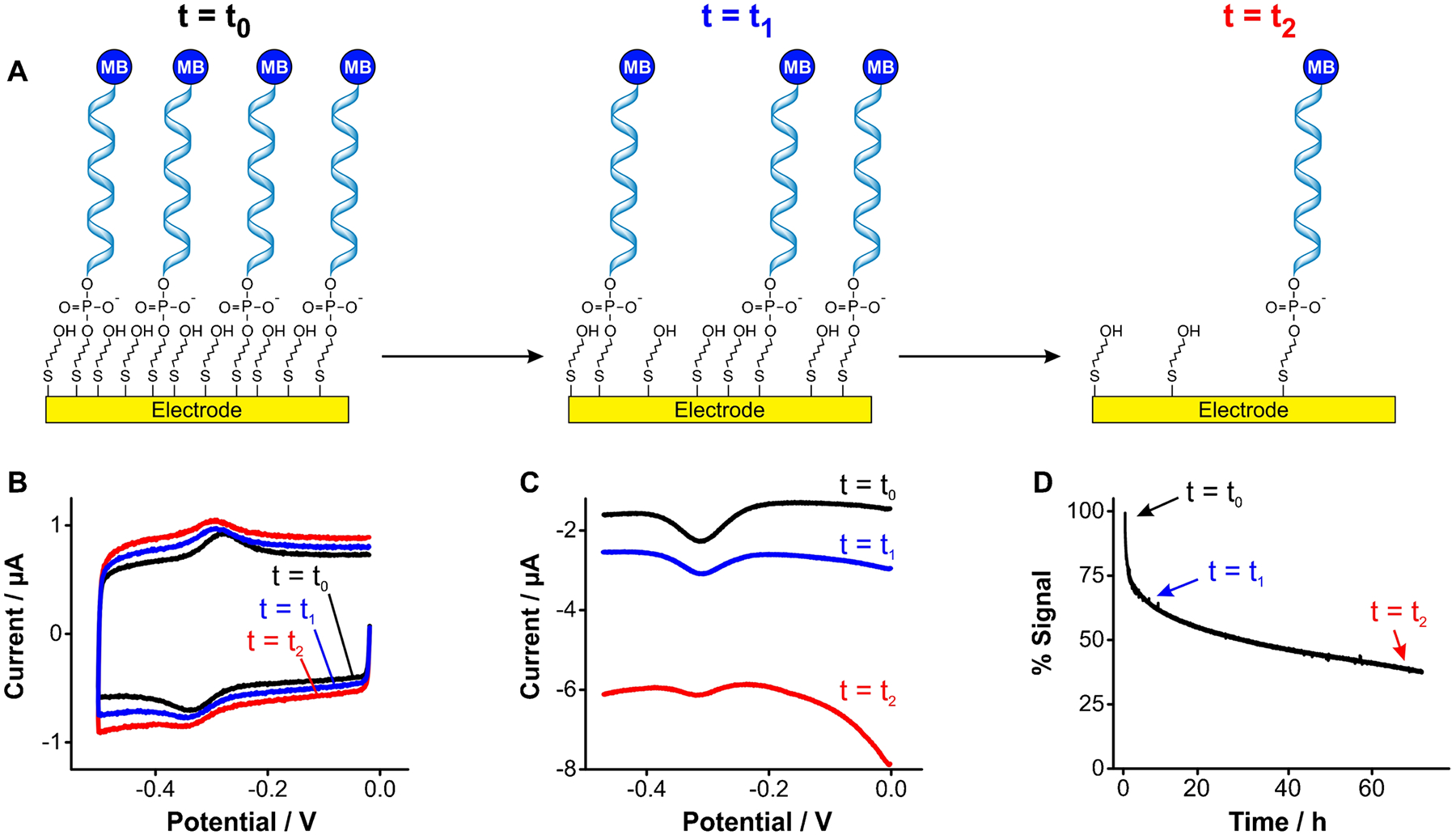
E-AB sensors undergo progressive signal loss over periods of hours. **A** Continuous voltage cycling of such sensors in causes progressive desorption of monolayer elements. **B** When we interrogate E-AB sensors using cyclic voltammetry, monolayer desorption is observed as a loss of redox currents from aptamer-bound methylene blue, in addition to an increase in charging and oxygen reduction currents. This is illustrated here via cyclic voltammograms measured from a tobramycin-binding E-AB sensor [18] at a scanning rate of 5 V s^−1^. **C** The same effects are observed when interrogating E-AB sensors via square wave voltammetry, namely, decreased magnitude of the redox peaks at ~ − 0.3 V and increased background from charging and oxygen reduction currents. Measurements performed using a square wave frequency of 300 Hz, amplitude of 50 mV, and step size of 1 mV. **D** Serially interrogating sensors by voltammetry results in a biexponential loss of signal over time

One key mechanism of signal loss in E-AB sensors is the progressive voltage-induced desorption of monolayer elements from the sensor’s gold surface (Fig. 2A), either by the voltage window used during the electrochemical interrogation or by the electrochemical technique used [8, 17]. This effect originates from the charged nature of DNA/RNA backbones, which can be electrostatically actuated (i.e., pushed away) by voltage biasing. Thus, upon continuous interrogation of E-AB sensors via cyclic voltammetry (Fig. 2B) or square wave voltammetry (Fig. 2C), E-AB signals decay over time.

On the one hand, the loss of methylene blue-modified aptamers translates into a decrease in redox currents. On the other hand, the loss of blocking alkanethiol molecules exposes the gold surface, increasing the capacitive current and enabling the reduction of dissolved oxygen (~ − 0.4 V), two effects that further decrease peak currents upon continuous interrogation (Fig. 2D). Such effects derive in part from the fact that thiol-gold bonds tethering aptamers to the gold surface of benchmark E-AB sensors are weak [19]. For example, using atomic force microscopy to measure surface-monolayer bond strength, measurements on thiol-gold interfaces reported forces of ~ 0.6 nN vs, for example, C-N bonds ~ 4.1 nN (a sevenfold difference) [20–23]. Moreover, monolayer assembly is known to weaken the thiol-gold bond strength [20], and thiol-gold monolayers are known to undergo hydrolytic desorption in physiological solutions (> 20% in 24 h) [24]. In addition, our group has previously demonstrated that the hydrophilicity of the terminal group of alkanethiols, which is needed to achieve strong E-AB signaling, further favors thiol-gold bond hydrolysis and desorption of monolayer elements from the E-AB surface [25]. Thus, a critical need remains to develop novel approaches to strengthen the monolayer-surface bond to overcome progressive degradation of E-AB sensing layers during continuous molecular measurements.

Several approaches have been reported in recent years to mitigate the E-AB signal decay caused by monolayer desorption under continuous electrochemical monitoring [16]. Some have focused on changing the chemical composition of the sensing layer, varying the nature of the blocking monolayer [25, 26], adding a second and silent redox reporter to the aptamer [27], or using a polymeric membrane that coats the electrode surface [7]. Others have focused on adjusting the electrochemical technique used for sensor interrogation, using drift-free approaches that extend sensors lifetime [11, 18], or mathematical methods that numerically correct signal loss in real time [28]. However, the utility of these strategies is ultimately limited by the weak nature of the thiol-gold bond. As an alternative to overcome this problem, here we explore the possibility of using carbon surfaces instead of gold to achieve covalent bonding of monolayer elements to the sensing electrode via carbon–nitrogen bonds, which can be fourfold stronger than thiol-gold bonds. We specifically investigated three different carbon surface modification strategies: surface anodization, grafting of arenediazonium ions, and grafting of primary aliphatic amines. We evaluated the ability of such approaches to form densely packed monolayers on the surface of glassy carbon (GC) electrodes and highlight the one strategy that can potentially serve as an alternative to thiol-gold chemistries. Using this strategy, we demonstrate its suitability to build DNA-based electrochemical sensors and we comparatively show improved surface stability in biological fluids for our carbon-based chemistry relative to benchmark thiol-gold interfaces.

## Materials and methods

### Chemicals and materials

Phosphate buffer saline (PBS, 11.9 mM HPO_3_^2−^, 137 mM NaCl, 2.7 mM KCl; pH 7.4), sodium chloride (NaCl), magnesium chloride hexahydrate (MgCl_2_·6H_2_O), sulfuric acid (H_2_SO_4_), sodium hydroxide (NaOH), new methylene blue (NMB), and guanidine hydrochloride were purchased from Fisher Scientific (Waltham, MA, USA). N-(3-dimethylaminopropyl)-N’-ethylcarbodiimide hydrochloride (EDC), N-hydroxysuccinimide (NHS), 4-aminobenzoic acid, hydrochloric acid (HCl), sodium nitrite (NaNO_2_), acetonitrile (ACN), ethanol (EtOH), sodium perchlorate (NaClO_4_), hexylamine, 6-amino-1-hexanol, 6-mercaptohexanoic acid, and potassium ferricyanide were purchased from Sigma-Aldrich (St. Louis, MO, USA). 6-Mercapto-1-hexanol, 1-hexanethiol, and 6-aminohexanoic acid were purchased from Alfa Aesar (Ward Hill, MA, USA). Tetrabutylammonium tetrafluoroborate (TBATFB) was purchased from TCI America (Seekon, MA, USA). All aqueous solutions were prepared in ultrapure deionized Milli-Q water. Gold working (cat. #002,314, Ø = 1.6 mm) and platinum counter electrodes (cat. # 012,961) were purchased from ALS (Tokyo, Japan). GC working (CHI104, Ø = 3.0 mm) and Ag/AgCl (1 M KCl) reference electrodes (CHI111) were purchased from CH Instruments (Austin, TX, USA). A ThermoMixer C from Eppendorf (Framingham, MA, USA) was used to carry out all the incubations and washes used for sensor fabrication.

A tobramycin-binding aptamer with a 6-carbon amine linker on the 5’ end and methylene blue on the 3’ end, with sequence 5’ H_2_N-(CH_2_)_6_ – GGG-ACT-TGG-TTT-AGG-TAA-TGA-GTC-CC – MB, was purchased from Integrated DNA Technologies (Coralville, IA, USA) and used as received. To enable the amine modification at the 5’ end, this company uses a longer linker than is conventionally found in aptamer papers (Fig. S1).

### Electrode cleaning and electrochemical measurements

GC electrodes were polished using a 1200/P2500 silicon carbide grinding paper (PN 36–08-1200, Buehler, USA), and a cloth pad with alumina slurry (PN CF-1050, BASi, USA). After this, they were sonicated in a 1:1 H_2_O/EtOH solution twice for 15 s. Gold electrodes were mechanically cleaned in the same way; however, additional electrochemical cleaning was performed by cyclic voltammetry, sweeping the voltage from − 0.3 to − 1.6 V in 0.5 M NaOH and from 0 to 1.6 V in 0.5 M H_2_SO_4_, 200 scans in each solution at a scan rate of 0.5 V/s, as previously reported [18]. A CH Instruments 8-channel potentiostat (CHI 1040C) was used for all electrochemical measurements. All voltage measurements referenced to Ag/AgCl electrodes. For the electrochemical characterization of the electrodes after each modification protocol, we recorded cyclic voltammograms in PBS and in a 5 mM ferricyanide solution prepared in PBS at a scan rate of 0.1 V/s. NMB-modified electrodes were characterized in PBS by cyclic voltammetry at a scan rate of 5 V/s, unless otherwise stated. Square wave voltammetry measurements were performed with an amplitude of 50 mV, a step size of 1 mV, and a frequency of 200 Hz. Continuous electrochemical interrogation of electrode surfaces was achieved via the use of macro scripts in CH software. We processed and visualized the resulting data using SACMES, a custom Python script previously reported by our group [29].

### Modification of gold electrodes with alkanethiols

To modify the surface of gold electrodes with self-assembled monolayers of short chain alkanethiols, we placed freshly polished and activated electrodes into 1 mM aqueous solutions of the different thiols (hexanethiol, mercaptohexanol, or mercaptohexanoic acid) for 2 h under constant stirring (1500 rpm), followed by rinsing with deionized water. Electrodes modified with mercaptohexanoic acid monolayers were further functionalized with the amine-containing redox reporter NMB (Fig. S2). For this, we immersed the electrodes in a solution containing 30 mM EDC, 15 mM NHS, 0.25 M NaCl, and 5 mM MgCl_2_ at pH = 6 for 2 h under constant stirring. After this, we rinsed the electrodes with water and immersed them into a 6 M solution of guanidine hydrochloride prepared in EtOH/H_2_O (4:1) for 5 min to remove non-specifically adsorbed NMB molecules from the surface. We used this coupling protocol in all methods studied in this work.

### Anodization of glassy carbon electrodes

For the anodization of GC electrodes, we adapted previously reported protocols for carbonaceous surfaces [30–32]. Briefly, we first anodized the electrodes at 1.75 V vs Ag/AgCl for either 30 or 300 s under constant nitrogen bubbling followed by a cathodization step at − 1.20 V vs Ag/AgCl for 10 s. After rinsing the electrodes with water, they were either used for the electrochemical characterization or functionalized with NMB via the EDC/NHS coupling reaction.

### Electrografting of arenediazonium ions

To graft in situ-formed arenediazonium ions on the surface of gold and GC electrodes, we followed a similar protocol to those previously reported by Bélanger and coworkers [33, 34]. Briefly, we immersed the electrodes in a 0.5 M HCl solution containing 2 mM NaNO_2_ and 1 mM aminobenzoic acid, followed by the electrochemical grafting of the generated arenediazonium ion via either linear sweep voltammetry or chronoamperometry. For the linear sweeps, we scanned the electrode voltage from 0.4 to − 0.2 V at a scan rate of 0.1 V/s. In the case of the electrografting by chronoamperometry, we polarized the electrodes at − 0.2 V for either 60 or 240 s. With both techniques, we circulated nitrogen through the cell during the electrografting step. After this, GC electrodes were rinsed with water and electrochemically characterized, before and after functionalization with NMB.

### Electrografting of aliphatic amines

For the grafting of aliphatic amines on the surface of GC electrodes, we followed a protocol like that reported by Barbier and coworkers [35]. We ran 20 cyclic voltammograms at 0.1 V/s from 0 to 1.75 V vs Ag/AgCl in a solution containing 10 mM of the selected amine and 0.1 M of TBATFB prepared in acetonitrile under constant stirring and continuous nitrogen bubbling. Due to the low solubility of aminohexanoic acid in acetonitrile, we used a 10 mM solution of this amine in ethanol containing 0.1 M of NaClO_4_ to carry out the electrografting [36]. After rinsing with deionized water, GC electrodes were ready for use.

### Nucleic acid immobilization on electrografted amines

Using GC electrodes modified with aminohexanoic acid as described above, we covalently coupled amine-modified aptamer molecules via reaction with EDC/NHS, like the approach we used to couple NMB. However, for aptamer coupling, we employed DNA solutions at a concentration of 1 μM in the presence of EDC/NHS (concentrations indicated above) for 5 h under constant stirring.

### Measurements in human serum

We performed monolayer stability measurements in undiluted human serum purchased from the vendor BioIVT (https://bioivt.com). The serum pool contained samples from 20 different individuals, ten females and ten males, mixed prior to shipping. The samples were obtained with consent by the commercial vendor and stripped of identity information at the site of storage prior to shipping. All human serum used in this work was destroyed immediately after use via incubation in 20% commercial bleach solution in water for 30 min, then discarded following Johns Hopkins University waste management procedures. All procedures employed in this work were approved by the Johns Hopkins University Biosafety Committee under protocol # B2004270102.

## Results and discussion

The side-by-side evaluation of carbon surface modification protocols strongly depends on the metrics used for the comparison. In this work, we were interested in evaluating whether the different surface functionalization strategies: (1) successfully formed a coating layer on the GC electrode surface, (2) the layer was sufficiently dense to dampen capacitive and oxygen reduction currents, and (3) the layer could be further functionalized to create sensing interfaces. To address the first point, a widespread approach in the field of electroanalysis is to evaluate the electron transfer between a solvated redox reporter and the electrode surface via cyclic voltammetry [37–39]. The idea is that, upon surface modification, electron transfer will be hindered to an extent that can be qualitatively correlated to the presence and density of the deposited layer. These measurements are typically performed in solutions containing potassium ferro/ferricyanide and/or hexaammineruthenium (III). As one can imagine, assessing a protocol’s extent of surface modification based exclusively on the measurement of electron transfer from these reporters can be misleading, since the reporters themselves have properties that can affect the determination. Here, in contrast, we followed a three-pronged approach to study the efficiency of surface functionalization of GC electrodes that directly reports on the three above-described metrics.

In our approach, we first measure the voltammetric response of ferricyanide following surface functionalization to qualitatively estimate the extent of electrode surface coverage. Because this estimation is highly sensitive to, for example, surface charge, we performed a second measurement in the same electrolyte (PBS) without ferricyanide to measure the effect of the surface modification protocol on capacitive and oxygen reduction currents [25]. This second determination allows us to confirm the presence of a coating layer and qualitatively estimate its packing density. The third evaluation consists of reacting free surface carboxylic groups resulting from the modification protocol with the redox reporter NMB via carbodiimide coupling reactions. This last step allows us to quantitatively determine the density of the coating layer and its ability to support further surface modification steps to allow the coupling of sensing elements.

### Alkanethiol-based monolayers on gold electrodes

We first illustrate our surface evaluation approach on gold electrodes, which serve as the benchmark reference for this work. To do this, we modified such electrodes with three different alkanethiols commonly used for E-AB sensing: hexanethiol, mercaptohexanol, and mercaptohexanoic acid [25, 40]. All these molecules consist of a 6-carbon aliphatic chain but have different terminal groups at the end opposite to the thiol-gold bond (–H, –OH, and –COOH, respectively). The chemical structure of such alkanethiols affects the packing density of the resulting monolayer, and thus the electrochemical response observed upon interrogation.

**Fig. 3:**
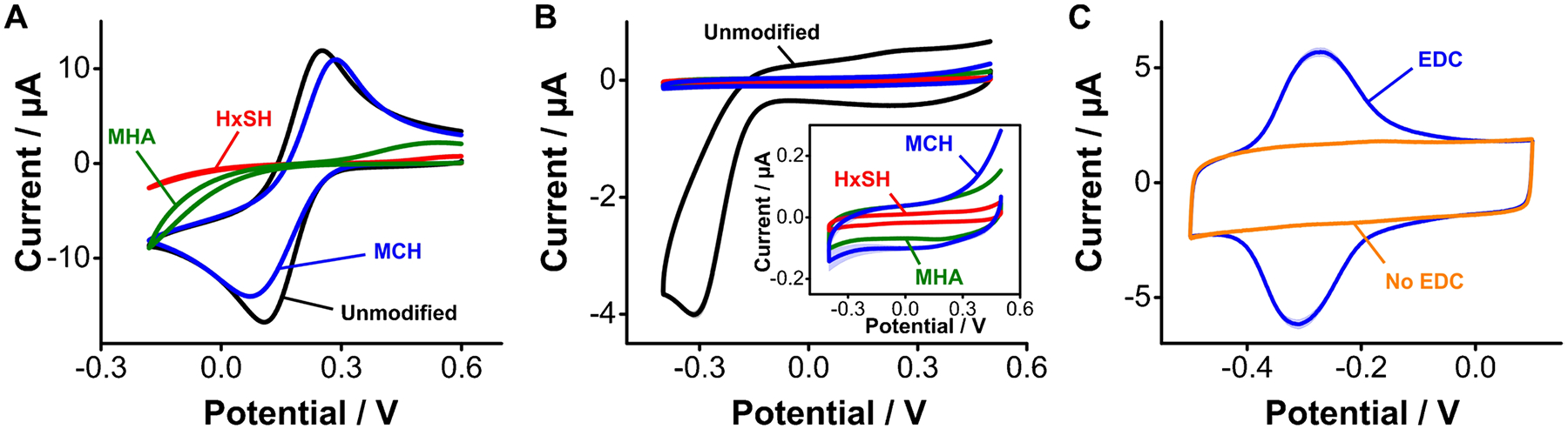
Electrochemical characterization of alkanethiol-based monolayers. **A** Assessment of through-monolayer electron transfer from ferricyanide. HxSH is hexanethiol, MCH is mercaptohexanol, and MHA is mercaptohexanoic acid. **B** Complementary measurement of capacitive and oxygen reduction currents in PBS. **C** The carbonyl groups present in a densely packed monolayer of mercaptohexanoic acid can be used to covalently attach redox reporters. Here, for example, we used the EDC/NHS chemistry to covalently attach NMB to them. The scan rates were 0.1 V/s for panels **A** and **B**, and 5 V/s for panel **C**. Each trace represents the average and shades the standard deviation of 4 electrodes

As mentioned above, using ferricyanide to probe the extent of carbon surface functionalization can be misleading, as the response of this reporter depends on surface functional groups. We illustrate this here by showing a side-by-side comparison of cyclic voltammograms measured on gold surfaces functionalized with monolayers of each of the three alkanethiols. Doing so we observed that, while electron transfer from ferricyanide to the gold surface was severely dampened by the presence of the hexanethiol monolayer, a much lesser effect is observed with mercaptohexanol, even though these alkanethiols form similarly dense monolayers (Fig. 3A–B) [25]. Likewise, strong dampening of the faradaic current from ferricyanide also occurred with mercaptohexanoic acid monolayers, which contain surface carboxylic groups that are negatively charged at the pH of our electrolyte (pH = 7.4). Electrostatic repulsion prevents ferricyanide molecules (which are negatively charged) from approaching the electrode surface to transfer electrons, dampening the net faradaic current regardless of packing density (which is approximately the same as that achieved by mercaptohexanol monolayers) [41]. Thus, the approach of measuring electron transfer from ferricyanide to determine the extent of surface coverage by a given monolayer can only be used qualitatively.

A complementary approach to study monolayer coverage involves the combined measurement of voltametric charging and faradaic currents from oxygen reduction. On the one hand, voltametric charging currents are a function of the electrode capacitance, which is strongly affected by monolayer thickness and organization [42]. On the other hand, molecular oxygen is sufficiently small to permeate the monolayer through pinholes or defects, thereby probing monolayer density with high fidelity. To illustrate these effects, we performed cyclic voltammetry on gold surfaces functionalized with hexanethiol, mercaptohexanol, and mercaptohexanoic acid in PBS in the absence of any solvated redox mediators. Doing so we observed a strong decrease in voltametric charging currents for all three monolayers relative to bare gold surfaces (Fig. 3B). In addition, we observed no faradaic current contributions from oxygen reduction (~ − 0.3 V). Expanding the *y*-axis of the voltammograms to better resolve the charging currents of monolayer-coated surfaces (inset in Fig. 3B), we observed that the monolayer packing achieved by hexanethiol is tighter than that of either mercaptohexanol or mercaptohexanoic acid (the capacitive current is lower), in agreement with the ferricyanide measurements. However, the charging currents from mercaptohexanol- or mercaptohexanoic acid-coated surfaces are within error of each other, indicating that these monolayers are similarly packed. These results indicate that the combined use of ferricyanide electron transfer and surface capacitive measurements provides a more accurate evaluation of the extent of surface functionalization.

Our third metric is to evaluate if a given surface functionalization protocol can support coupling of biosensing elements (i.e., reporter-modified aptamers), as required to produce E-AB sensors. On gold surfaces, E-AB sensors are fabricated via co-incubation in solutions containing blocking alkanethiols plus alkanethiol- and redox reporter-modified aptamers to form mixed self-assembled monolayers. However, on carbon surfaces, the coupling of aptamers as required for E-AB sensing will necessarily be a second step that follows surface functionalization. For example, in this work, we evaluate the ability of three carbon functionalization strategies to support carbodiimide coupling between surface carboxylic groups and the amine-containing redox reporter NMB. As a reference for our work, we first performed this characterization on gold surfaces functionalized with mercaptohexanoic acid monolayers, which contain carboxylic groups available to coupling reactions. Voltammograms measured after treating these monolayers with NMB in the absence or presence of EDC/NHS show functionalization of the monolayer only when the EDC reagent is present (Fig. 3C). We note that we coupled the reporter NMB to the various carbon surfaces directly, without the aptamer. This is a cost-saving measure that allows us to characterize the carbon surface under various conditions without wasting aptamer moles. However, aptamers can be purchased from vendors with terminal amine or activated ester modifications that readily enable carbodiimide coupling reactions, as we show below.

### Surface layers based on anodization of glassy carbon electrodes

**Fig. 4:**
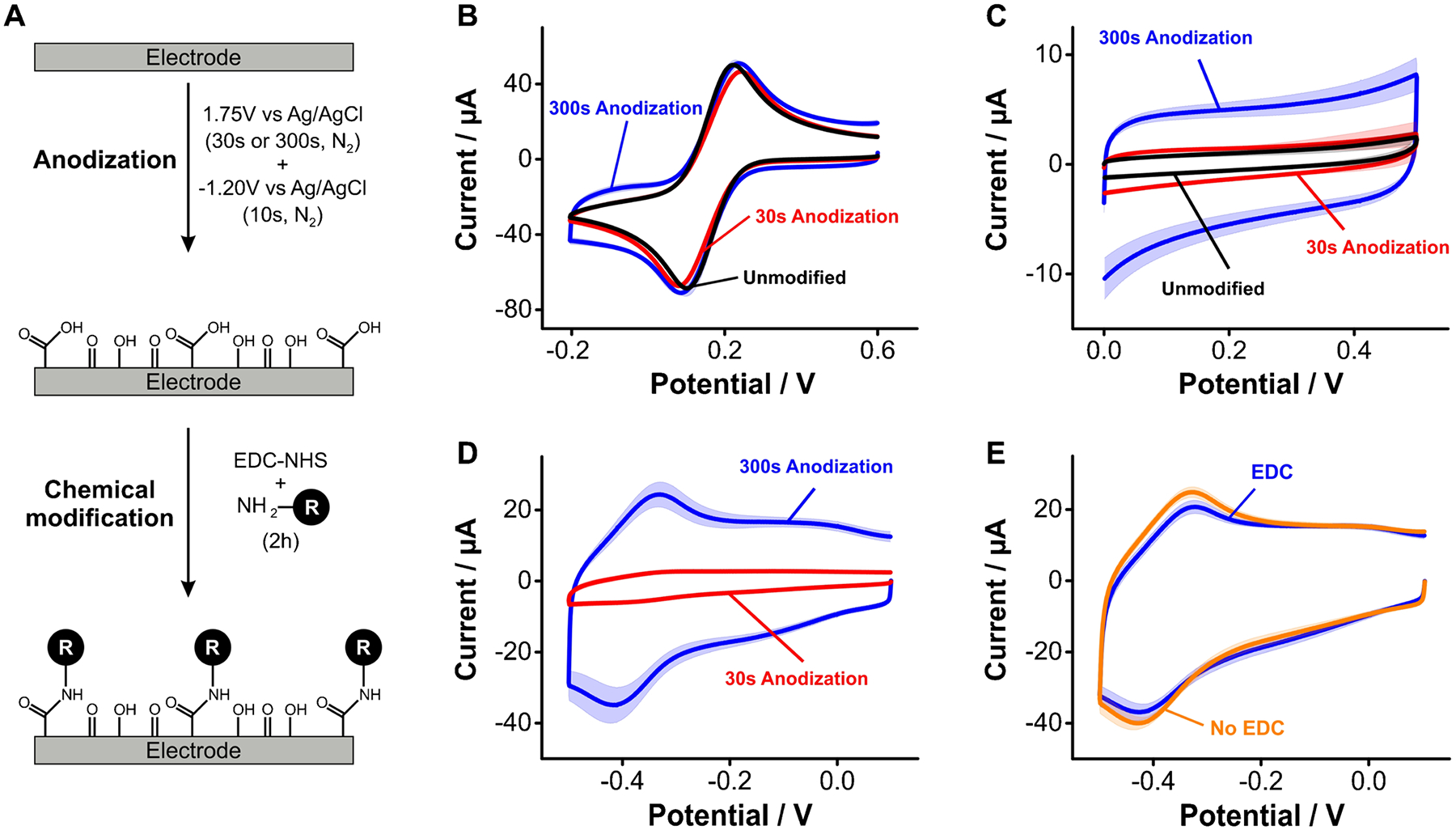
Anodization of GC electrodes to introduce surface carboxylic acid groups. **A** Protocol for anodization of GC electrodes followed by EDC/NHS-mediated coupling of the redox reporter NMB. **B** Cyclic voltammograms obtained after anodization in ferricyanide solutions show no discernible changes in electron transfer (i.e., voltammogram shape) post surface treatment. **C** However, electrode anodization leads to a significant increase in charging currents from PBS solutions proportional to anodization time. **D** Exposure of anodized GC surfaces to NMB and EDC/NHS results in the appearance of forward and reverse redox waves at ~ − 0.4 V. **E** However, control measurements in the absence of EDC/NHS show a similar voltammetric response, indicating that the redox processes at − 0.4 V correspond to non-specifically adsorbed NMB molecules on the GC electrode surface. All the voltammograms were measured at a scan rate of 0.1 V/s. Solid traces and shaded areas represent the average and standard deviation, respectively, from 4 electrodes

We evaluated the possibility to generate carboxylic acids on the surface of GC electrodes via anodization to enable chemical coupling of primary amines via carbodiimide reactions. The idea was to create monolayers of aliphatic groups using amide bonds. We specifically tested two previously published protocols [30–32] (Fig. 4A): one using a short anodization period in which electrodes were polarized at 1.75 V vs Ag/AgCl for 30 s, and a second one with a longer anodization period of 300 s at the same voltage bias. Assessing the resulting carbon surfaces via electron transfer from ferricyanide solutions indicated virtually no surface functionalization with carboxylic acids (i.e., no changes in voltammogram shape, Fig. 4B). In contrast, we observed a definitive increase in charging currents following anodization for 300 s (Fig. 4C). This increase in capacitive charging was coupled to the formation of a carbonaceous layer on the electrode surface, distinctively visible by the naked eye as debris that formed during electrode polishing post surface treatment. We speculate this layer is the product of erosion of the carbon surface caused by both aggressive oxidation and oxygen bubbling from water oxidation occurring at 1.7 V. Thus, the increase in charging currents likely reflects an increase in surface area caused by the formation of pores in the GC electrode [43].

Chemical coupling of amine-modified redox reporters to anodized carbon surfaces did not occur in our hands. Instead, we observed a significant amount of physiosorbed reporter stuck within the porous structure of the carbon layer produced by anodization. We illustrate this effect by first showing voltammograms of GC electrodes after a 30 s (red trace) or 300 s (blue trace) anodization and chemical coupling to NMB via carbodiimide reaction (Fig. 4D). The coupling reaction produced the appearance of forward and reverse redox waves at − 0.4 V (~ 100 mV more negative than on gold, Fig. 4D vs Fig. 3C). We observed similar effects but much lower in magnitude after a 30-s-long anodization. However, the NMB redox waves were visible even in the absence of the EDC/NHS reagents (Fig. 4E), an indication that the coupling reaction did not occur. Instead, such redox waves likely arise from non-specific binding of NMB to the carbon surface, presumably via π–π stacking interactions with aromatic groups, a well-documented effect [44, 45].

Overall, our combined results using surface anodization strategies did not offer a promising path for the practical functionalization of carbon surfaces for E-AB sensing applications, at least within the limits of the two strategies we tested here. Seeking to eliminate the erosion of carbon electrodes and achieve direct attachment of functional groups on their surface, we next explored the electrografting of arenediazonium ions and aliphatic amines.

### Electrochemical grafting of diazonium salts

**Fig. 5:**
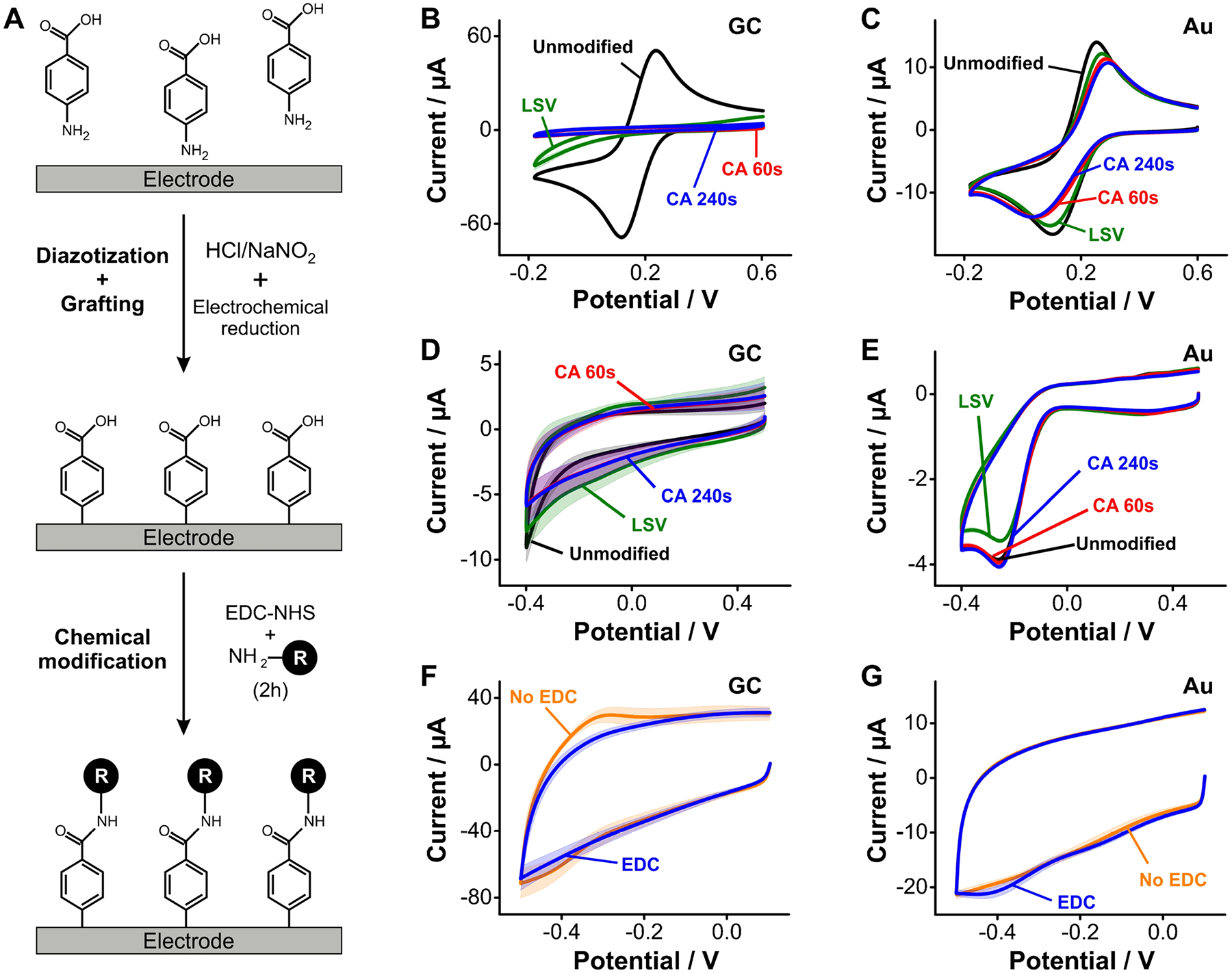
Electrochemical grafting of arenediazonium ions. **A** This work used aminobenzoic acid to generate an arenediazonium ion in situ in the presence of HCl and NaNO_2_. The resulting layer contained free carboxylic groups available for EDC/NHS coupling of the reporter NMB. **B** Grafting of arenediazonium ions strongly affected electron transfer from ferricyanide to GC electrodes. **C** In contrast, monolayers produced via the same protocol on gold electrodes only partially affected electron transfer from ferricyanide. **D**, **E** Arenediazonium grafting onto GC and gold electrodes changed charging and oxygen reduction currents with a similar trend as the one observed from ferricyanide solutions. **F**, **G** Coupling of NMB to the free carboxylic acids from arenediazonium layers was unsuccessful in our hands. Instead, we observed redox waves that again correspond to NMB non-specifically bound to the electrode surfaces. Solid lines and shades correspond to the average and standard deviation of 4 electrodes, respectively

Ex situ- or in situ-generated arenediazonium ions can be electrografted onto electrode surfaces by applying a reducing voltage of < 0.1 V vs Ag/AgCl (Fig. S3), generating a covalent bond between a carbon atom in their aromatic ring and the electrode surface. To evaluate the packing and organization of the molecular layers resulting from this method, we generated diazonium ions of aminobenzoic acid in situ and electrografted them to the surface of GC and gold electrodes (Fig. 5A). Attempting to mitigate the formation of multilayers [46], we evaluated three different protocols to control the extent of electrode surface modification: first, we used linear sweep voltammetry applying a single cathodic scan to limit multilayer growth; second, we tried time-modulated chronoamperometry, polarizing the electrodes at a reducing voltage for 60 s; and third, we performed the same chronoamperometry but for 240 s. Initial evaluation of linear sweep voltammograms in ferricyanide solutions showed a strong passivation of the GC (Fig. 5B) but not the gold electrodes (Fig. 5C). In addition, chronoamperometry-based grafting produced the most electrode coverage on GC electrodes based on this measurement.

Determining the charging and oxygen reduction currents post grafting of arenediazonium ions on GC and gold electrodes produced similar results to those observed via ferricyanide measurements. Specifically, oxygen reduction currents decreased by a factor of two from bare GC to arenediazonium-coated electrodes (Fig. 5D). In contrast, we did not observe a change in oxygen currents for gold surfaces (Fig. 5E), relative to untreated electrodes. These results may suggest that GC surfaces allow for higher density grafting of arenediazonium ions than gold. However, the extent of surface coverage for both electrode types is clearly low, causing only minor differences in capacitive currents. Further, attachment of NMB to the grafted benzoic acid groups via EDC/NHS coupling did not result in observable redox waves in cyclic voltammetry. Instead, we again observed non-specific binding of the reporter to the carbon surfaces, which in this case seemed to be mitigated by the presence of the EDC/NHS reagents (Fig. 5F). On gold, we observed the appearance of a small reduction wave at ~ − 0.4 V following the coupling reaction, but no reverse wave to indicate that NMB reporters were bound to the surface in significant amounts. These last results confirm that arenediazonium groups were grafted onto both carbon and gold surfaces, but the extent of the reaction and electrode coverage were too low to produce functional monolayers for E-AB sensing purposes.

### Electrochemical grafting of primary aliphatic amines

**Fig. 6:**
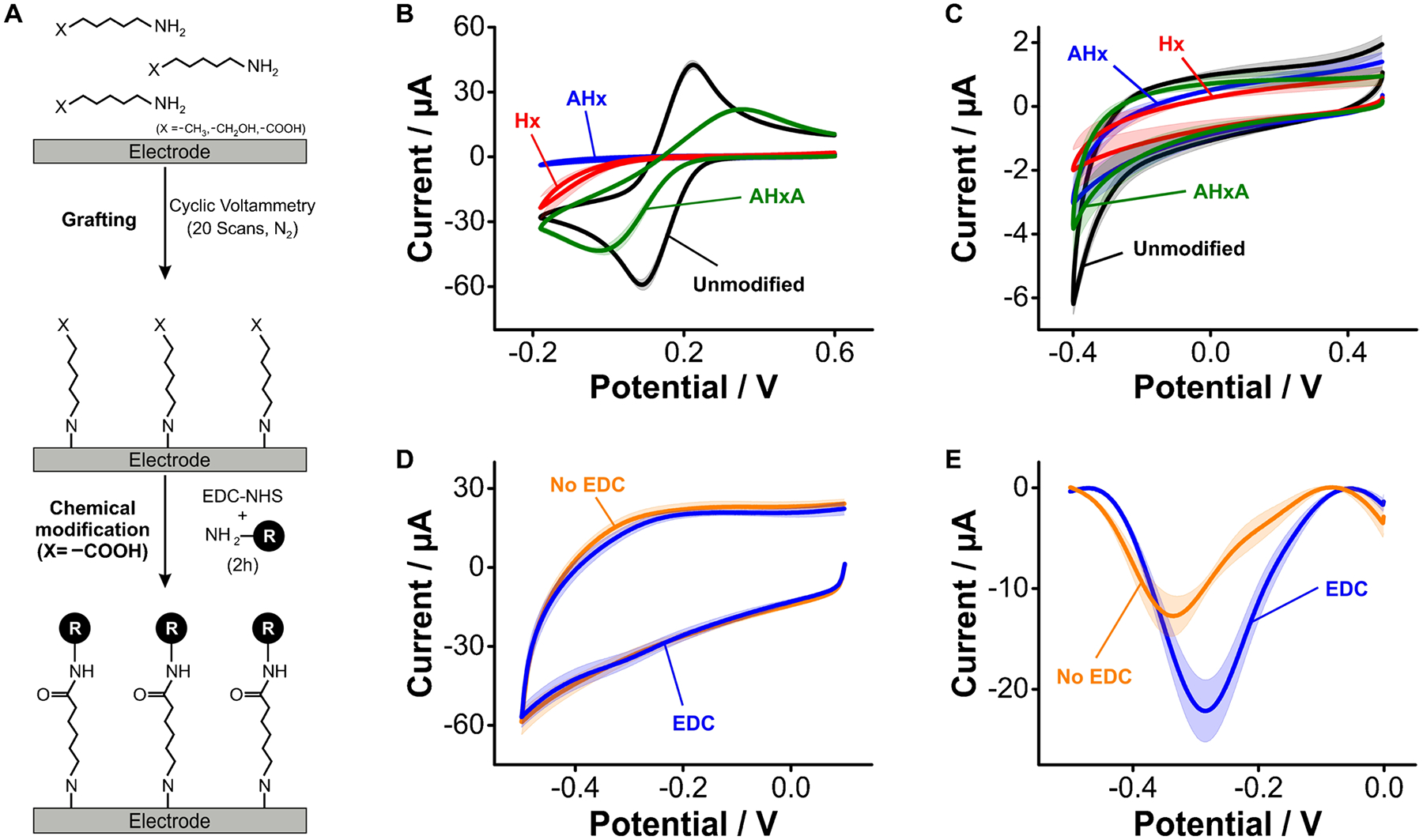
Electrochemical grafting of primary aliphatic amines. **A** Here, we employed the primary aliphatic amines aminohexane (Hx), aminohexanol (AHx), and aminohexanoic acid (AHxA). These were grafted onto GC electrodes by applying a sufficiently positive voltage to oxidize the primary amine and form C-N bonds, leading to the formation of a monolayer. **B** Through-monolayer electron transfer from ferricyanide was significantly hindered by electrode coating with all three primary aliphatic amines, **C** observing a similar trend when looking at charging and oxygen reduction currents in PBS. **D** When we coupled NMB to the free carboxylic groups present in AHxA layers, we could only observe a minimal difference in cyclic voltammetry from molecules non-specifically bound to the electrode surface. **E** In contrast, using square wave voltammetry, we were able to distinguish populations of covalently bound vs physisorbed NMB molecules. Solid traces and shades correspond to the average and standard deviation of 4 electrodes, respectively

The nitrogen atom of primary aliphatic amines can be oxidized on GC electrodes at voltages > 1.2 V vs Ag/AgCl to generate surface C-N bonds that lead to the formation of monolayers (Fig. S4). To illustrate this process, here we used cyclic voltammetry to electrograft three primary amines consisting of a 6-carbon chain with different terminal groups (aminohexane –H, aminohexanol –OH, and aminohexanoic acid –COOH, Fig. 6A). We selected these aliphatic amines to have a direct comparison against hexanethiol, mercaptohexanol, and mercaptohexanoic acid monolayers on gold electrodes (Fig. 3). Following the electrografting step, we observed significant dampening of ferricyanide redox currents caused by the formation of dense monolayers from the three amines evaluated (Fig. 6B). In the case of aminohexane and aminohexanol, we observed a complete blockage of electron transfer from ferricyanide. In contrast, electrografting of aminohexanoic acid did not achieve complete dampening of ferricyanide’s electron transfer, an indication that such a carboxylated amine did not form dense monolayers. Likewise, the measurement of charging and oxygen reduction currents following electrografting (Fig. 6C) revealed that aminohexane and aminohexanol achieved an ~ threefold decrease in charging currents (within error from each other), and complete removal of the oxygen reduction reaction. Electrodes functionalized with aminohexanoic acid, in contrast, achieved lesser surface coverage, thereby presenting charging and oxygen reduction currents closer to those observed on bare GC electrodes.

Carbodiimide coupling of the carbonyl groups from electrografted aminohexanoic acid with NMB successfully resulted in the appearance of forward and reverse redox waves at ~ − 0.3 V (Fig. 6D). This is the same reduction potential seen on gold electrodes functionalized with mercaptohexanoic acid monolayers (Fig. 3C). However, such redox waves were comparable in magnitude to background charging currents. Thus, to better resolve the success of the coupling reaction, we used square wave voltammetry, a differential technique that removes double layer charging while enhancing faradaic currents (Fig. 6E). Square wave voltammograms of the surfaces functionalized with EDC revealed a large redox wave corresponding to the reduction of electrode-bound NMB molecules. In contrast, control voltammograms of aminohexanoic acid monolayers exposed to NMB in the absence of EDC showed redox waves corresponding to non-specifically bound NMB displaying more negative reduction potentials (~ − 0.4 V), like those seen from anodized (Fig. 4E) and arenediazonium-functionalized (Fig. 5F) GC surfaces, as well as lower currents.

Our combined results so far indicate that electrografting of aliphatic amines onto GC surfaces is a good strategy to produce on-carbon blocking monolayers. The surfaces obtained through this method achieved effects on electron transfer from ferricyanide, charging and oxygen reduction currents, and packing densities (Fig. S5) that closely compared to those produced by alkanethiol self-assembly on gold electrodes. Motivated by such results, we next explored the suitability of electrografted amines to support the chemical immobilization of DNA molecules.

### Aptamer coupling to aminohexanoic monolayer on carbon

**Fig. 7:**
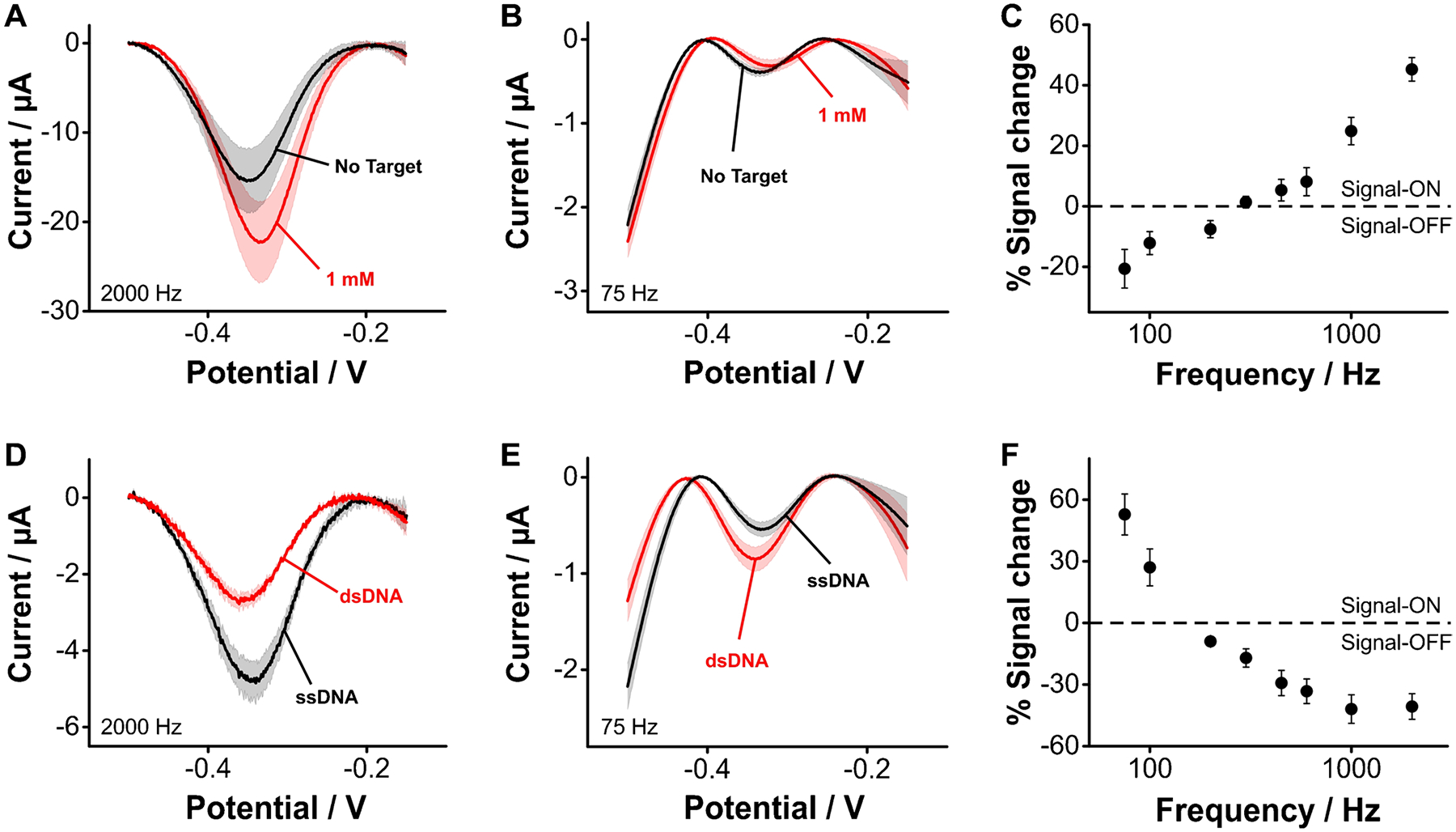
Monolayers produced on carbon via electrografting of aliphatic amines allow covalent coupling of aptamers to support sensing of small molecules and complimentary DNA strands. We illustrate this by coupling tobramycin-binding aptamers to electrografted monolayers of aminohexanoic acid, and challenging the resulting sensors with saturating target concentrations. First, to demonstrate E-AB sensing of small molecules, we challenged the sensors with tobramycin, observing (**A**) ON and (**B**) OFF responses that depended on square wave frequency, a behavior that is similar to that of benchmark, gold-based E-AB sensors. **C** Frequency map showing signal change calculated as the difference between sensors exposed to saturating tobramycin concentrations (1 μM) and sensors in blank buffered solutions. Second, we demonstrated sensing of DNA hybridization. We challenged our carbon-based sensors with saturating concentrations (10 μM) of DNA strands fully complementary to our aptamer sequence. Doing so we observed sensor (**D**) OFF and (**E**) ON responses that depended on square wave frequency. **F** Frequency map showing signal change calculated as the difference between sensors exposed to fully complementary DNA and sensors in blank buffered solutions. All measurements show the average of *n* = 4 sensors and corresponding standard deviations

Grafted amines with a functional group at the opposite end of the N–C bond can be used to attach biorecognition elements on the electrode surface, thus enabling the fabrication of electrochemical sensors. Here, we illustrate this by attaching tobramycin-binding aptamer molecules to the carboxylic groups of electrografted aminohexanoic acid via carbodiimide coupling (Fig. S6), under conditions similar to those employed for the coupling of NMB. We challenged the resulting aptamer-based sensors with tobramycin at different square wave frequencies to reveal the ON and OFF signaling that is expected from E-AB sensors. At square wave frequencies ~ 2000 Hz, the presence of tobramycin at a 1 mM concentration increased the sensor current output (Fig. 7A). In contrast, at frequencies ~ 75 Hz, the sensor current decreased (Fig. 7B). This square wave frequency dependence is better visualized using frequency maps (Fig. 7C), which depict sensor gain as the difference in voltammetric peak currents when the sensors are exposed to saturating target concentration relative to the absence of target. Building such maps, we observe OFF signaling at frequencies < 200 Hz and strong ON signaling at faster frequencies. These results highlight the ability of electrografted monolayers to support conventional E-AB sensing. However, the approach can be generalized to other sensing schemes that depend on the availability of surface-bound DNA to achieve detection. For example, we assessed the ability of our approach to support recognition of DNA hybridization. For this, we interrogated our carbon-based sensors before and after incubating them in solutions containing DNA strands fully complementary to our aptamer sequence, for 30 min. Doing so, we observed frequency dependent OFF (Fig. 7D) and ON (Fig. 7E) responses, which could also be mapped via a frequency sweep (Fig. 7F). Thus, this approach is generalizable and supports other DNA-based sensing strategies and can potentially be used to achieve sensing using biorecognition elements other than DNA (peptides, proteins, glycans, etc.).

### Monolayer stability under continuous voltammetric cycling

The carbon–nitrogen chemical bond resulting from electrografting of primary amines should be stronger than the thiol-gold bond. Thus, we anticipated this functionalization strategy should allow us to build monolayer-supported biosensing interfaces enabling stable voltammetric interrogation for longer periods of time relative to benchmark alkanethiol monolayers. To evaluate this hypothesis, we prepared two electrode batches, one consisting of GC electrodes electrografted with aminohexane, and the second comprising of gold electrodes coated with hexanethiol. These two systems are analogous in chemical functionality, except for the underlying surface and bond type. We then immersed these electrodes in a PBS solution and simultaneously interrogated them by cyclic voltammetry, every ~ 10 s for 48 h. Doing so allowed us to indirectly visualize the loss of monolayer groups from each electrode surface over time, by proxy of monitoring changes in charging and oxygen reduction currents.

**Fig. 8:**
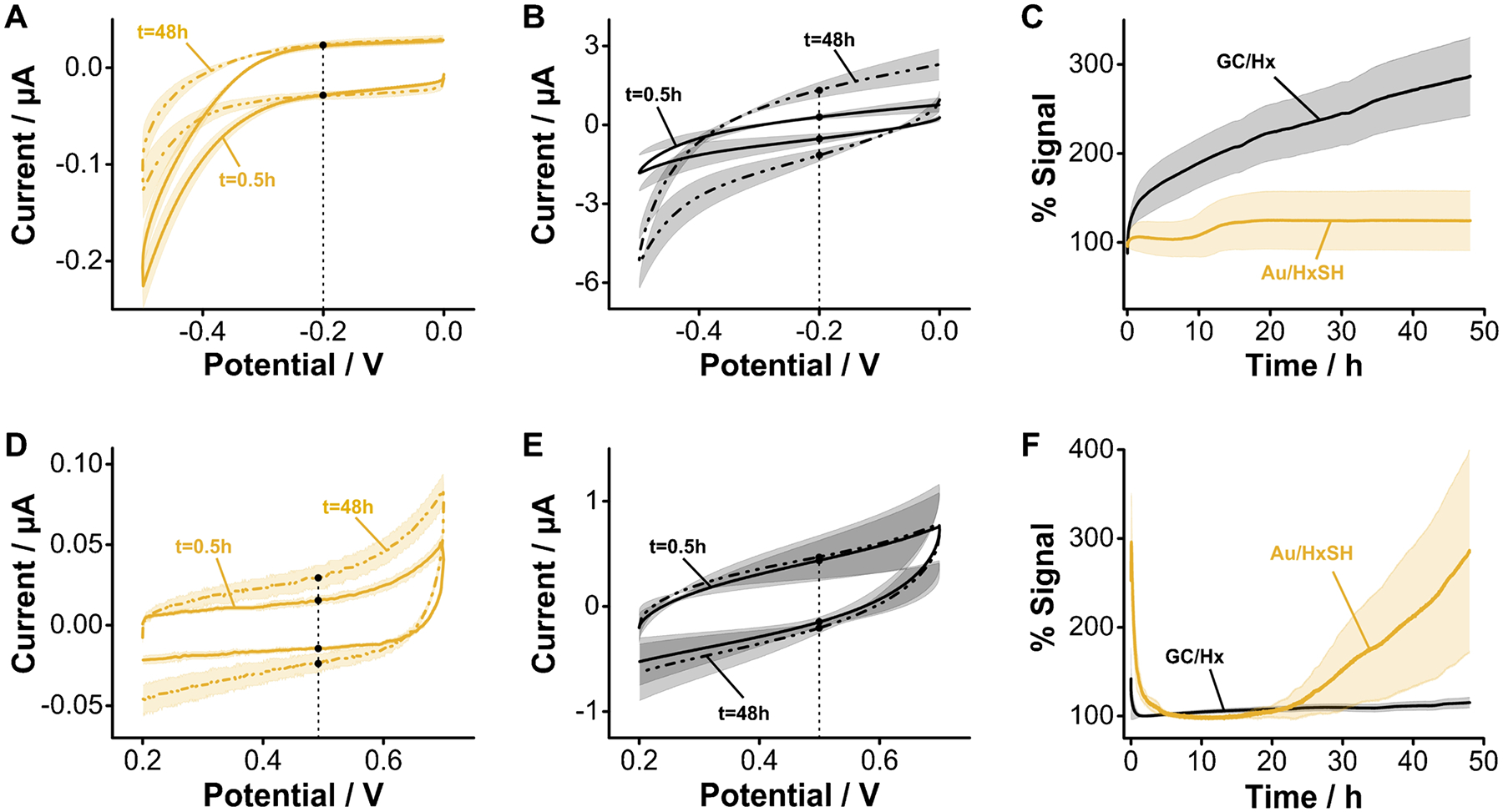
Monolayer stability under continuous interrogation. We first interrogated the electrodes negatively using a voltage window similar to the one used with benchmark E-AB sensors. **A** Gold electrodes modified with hexanethiol (HxSH) show almost no change in charging current after 48 h of continuous interrogation. **B** In contrast, GC electrodes modified with aminohexane (Hx) show a clear increase in charging current due to the reduction and removal of the grafted amine groups. **C** Side-by-side comparison of changes in charging currents measured at − 0.2 V for both sets of electrodes. **D** Interrogating positively, alkanethiol-coated gold electrodes presented a significant increase in charging currents. **E** In contrast, the charging currents of aminohexane-grafted GC electrodes were stable for 48 h. **F** Side-by-side comparison of charging currents vs time showing that, in this voltage window, aminohexane-grafted GC electrodes display a far superior stability. Solid, dotted traces, and shades correspond to the average and standard deviation of 4 electrodes, respectively

The voltammetric stability of GC surfaces electrografted with aliphatic amines when cycling between 0.0 and − 0.5 V vs Ag/AgCl is worse than that achieved by alkanethiols on gold. This voltage window is relevant because it covers the range typically employed to interrogate DNA biosensors functionalized with methylene blue reporters. The voltammograms measured from hexanethiol-functionalized gold surfaces (Fig. 8A) in this voltage range displayed a negligible change in charging currents (~ 15%) after 48 h of continuous interrogation (~ 18,000 scans). In contrast, voltammograms measured from GC electrodes electrografted with aminohexane presented a threefold increase in charging currents during the same period (Fig. 8B). This is an indication that the blocking monolayer was progressively removed during voltammetric cycling, presumably because the low voltage limit was sufficiently negative to cleave carbon–nitrogen bonds. When comparing the charging currents obtained from both batches of electrodes side-by-side (Fig. 8C), it becomes evident that while the alkanethiol-gold monolayers remained stable during the entire interrogation period, the GC-electrografted monolayers were progressively cleaved.

The stability trend reverses, however, when we voltammetrically cycle the electrodes in the positive direction, from 0.2 to 0.7 V vs Ag/AgCl. In this direction, the gold electrode surface can be oxidized at voltages above 0.2 V, producing significant changes in voltammetric charging currents (Fig. 8D). However, the carbon–nitrogen bonds resulting from aliphatic amine electrografting do not get electrochemically reduced anymore, thereby producing stable voltammograms even after 48 h (~ 13,000 scans) of continuous voltammetric cycling (Fig. 8E). The side-by-side comparison of charging currents over time (Fig. 8F) unequivocally demonstrates that in this voltage range, aminohexane-coated GC electrodes achieve far superior stability over alkanethiol monolayers on gold. These results highlight a promising path for the development of biosensors using redox reporters having a positive reduction potential.

**Fig. 9:**
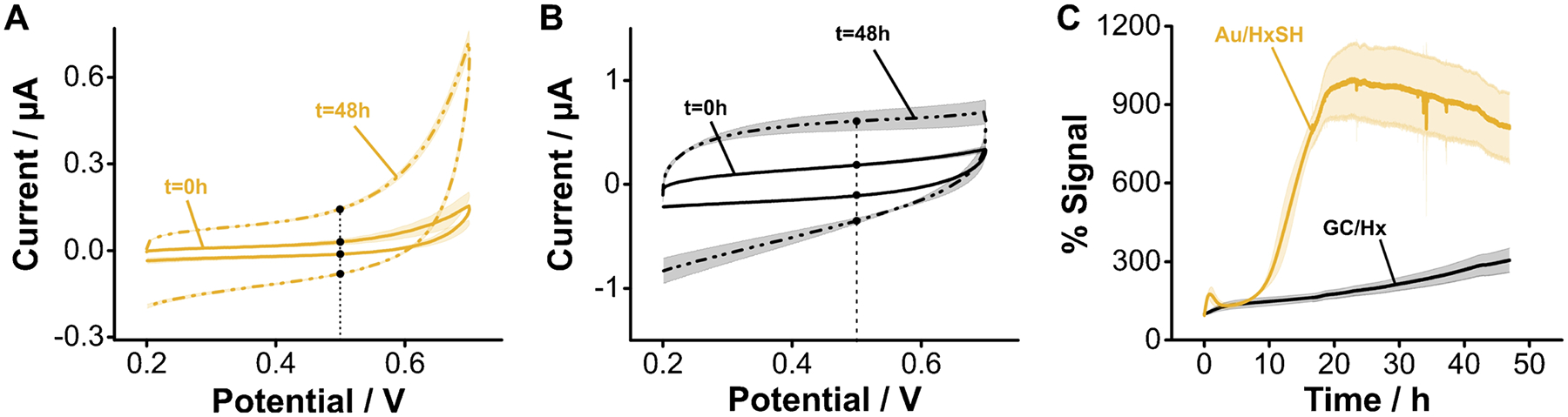
Monolayer stability under continuous interrogation in undiluted serum. **A** The voltammetric response of gold electrodes modified with hexanethiol shows a complete loss of the monolayer after 48 h of continuous interrogation. **B** In contrast, GC electrodes modified with aminohexane show less drift. **C** The side-by-side comparison of changes in charging currents measured at 0.5 V for both sets of electrodes under continuous interrogation in undiluted serum shows that after ~ 20 h, the HxSH monolayers are completely lost, while Hx monolayers experience significantly less degradation. Solid, dotted traces, and shades correspond to the average and standard deviation of 4 electrodes, respectively

The superior stability of aminohexane monolayers on GC is retained when the electrodes are interrogated in biological fluids such as undiluted human serum. This matrix contains small molecule thiols and thiolated proteins that can competitively displace alkanethiol monolayers from gold surfaces. However, the carbon–nitrogen bond achieved via electrografting does not undergo competitive displacement. Here we demonstrate this effect by comparatively evaluating the relative change in charging currents between alkanethiolfunctionalized gold and aminohexane-grafted GC electrodes during continuous voltammetric interrogation (Fig. 9). In undiluted human serum, alkanethiol monolayers start to appreciably desorb from gold surfaces after 7 h. Voltammograms measured after 48 h show a complete loss of blocking monolayer from the gold electrodes (Figs. 9A,C and S7A). However, during the same period, voltammograms of aminohexane-functionalized GC electrodes showed negligible changes in charging currents (Figs. 9B,C and S7B). These promising results will motivate future research in our group focused on demonstrating optimized coupling of aptamers to the GC-functionalized surfaces and the development of redox reporters with positive reduction potentials that enable continuous E-AB sensing for extended periods of time.

## Conclusions

We evaluated the possibility of using surface functionalization strategies to develop carbon surfaces amenable to E-AB sensing applications. For this purpose, we tested three protocols producing surface carboxylic acids for posterior carbodiimide coupling of primary amines: electrode anodization, grafting of arenediazonium ions, and primary aliphatic amines. Our results indicate that the electrografting of aliphatic amines is the only surface functionalization strategy achieving interfacial electron transfer, charging currents, and efficiency of surface coupling reactions that compare well to the self-assembly of alkanethiols on gold. Using monolayers produced by electrografting of aliphatic amines, we demonstrate the successful coupling of one aptamer sequence to the carbon-based sensors, enabling E-AB sensing of the small molecule tobramycin, and detection of a hybridization event using a DNA sequence fully complementary to the aptamer. The success of these experiments highlights the potential of this approach to be generalizable to other electrochemical sensing strategies requiring covalent coupling of biorecognition elements to carbon surfaces. Although the stability of the reported aminohexane monolayers on GC electrodes is compromised when sweeping the voltage negative where the reduction of methylene blue occurs, these monolayers are more stable than alkanethiol monolayers on gold when sweeping in the positive direction. Future optimizations of the amine grafting protocol and the interrogation voltage window may further extend the stability of amine-grafted GC surfaces. Thus, building from this work, future efforts from our laboratory will focus on further optimizing the coupling of amine-modified aptamers to enable robust E-AB sensing. We are also working towards the development of redox reporters with a positive reduction potential for use on GC-supported E-AB sensors.

## Supplementary Material

Supplementary Information
