# Supplementary material for: Study of surface modification strategies to create glassy carbon-supported, aptamer-based sensors for continuous molecular monitoring
Source: Anal Bioanal Chem. Author manuscript; Available in PMC 2022 Jul 1. (PMC9242903; doi:10.1007/s00216-022-04015-5)
Supplement: Supplementary Information [file NIHMS1802220-supplement-Supplementary_Information.docx]

**SUPPLEMENTARY INFORMATION**

**Study of Surface Modification Strategies to Create Glassy Carbon-supported, Aptamer-based Sensors for Continuous Molecular Monitoring**

Miguel Aller Pellitero^1*^ and Netzahualcóyotl Arroyo-Currás^1,2,*^

^1^ Department of Pharmacology and Molecular Sciences, Johns Hopkins University School of Medicine, Baltimore, Maryland, 21205

^2^ Department of Chemical and Biomolecular Engineering, Johns Hopkins University, Baltimore, Maryland, 21218

Correspondence to:

Miguel Aller Pellitero, Ph.D.

Johns Hopkins University School of Medicine

Department of Pharmacology and Molecular Sciences

Hunterian Building, Room 314

725 North Wolfe St.

Baltimore, MD 21205

mapellitero@jhmi.edu

Netz Arroyo, Ph.D.

Johns Hopkins University School of Medicine

Department of Pharmacology and Molecular Sciences

Hunterian Building, Room 316

725 North Wolfe St.

Baltimore, MD 21205

netzarroyo@jhmi.edu

(443) 287-4798

| \| **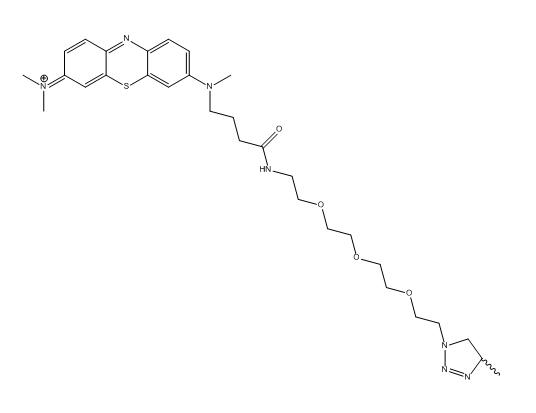** \| \| --- \| \| **Fig. S1** Structure of the linker used by Integrated DNA Technologies to attach methylene blue moieties to the terminal end of amine-terminated DNA sequences. \| |
| --- | --- | --- |
|  |

| **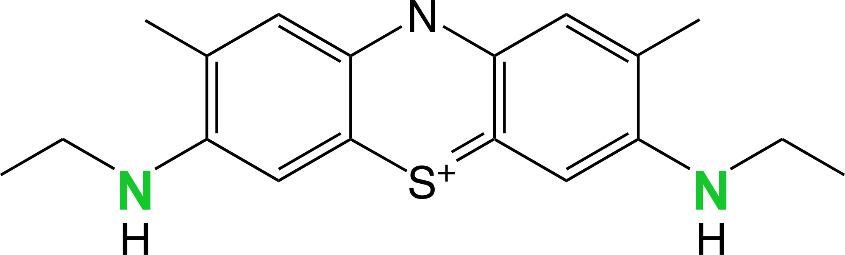** |
| --- |
| **Fig. S2** Molecular structure of New Methylene Blue (NMB). Unlike conventional Methylene Blue, NMB has two secondary amines (highlighted in green) that we use for EDC/NHS coupling to surface carboxylic groups. |

| **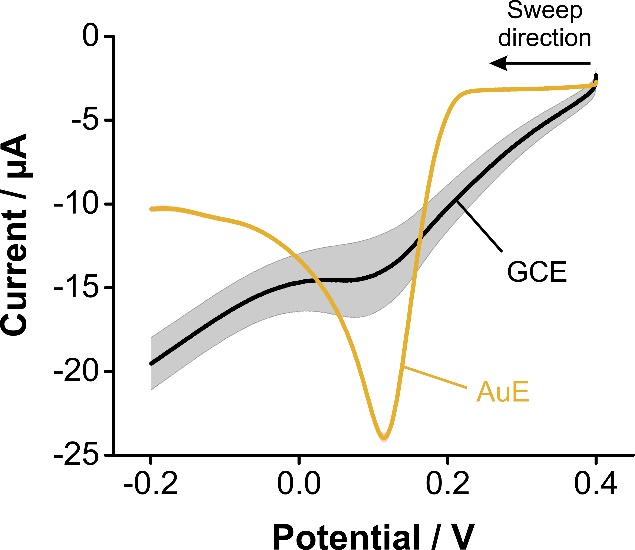** |
| --- |
| **Fig. S3** Linear sweep voltammograms obtained during the electrografting of arenediazonium salts. Each voltammogram represents the average and standard deviation of 8 electrodes. Scan rate of 0.1 V/s. Electrolyte: 0.5 M HCl solution containing 2 mM NaNO2 and 1 mM aminobenzoic acid. |

| **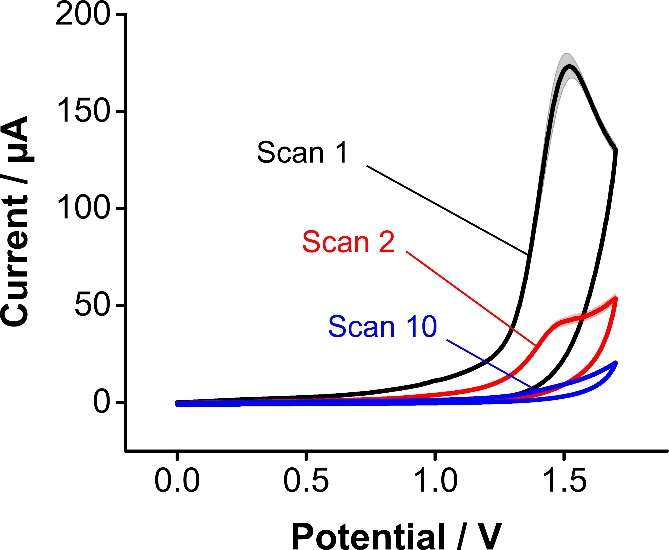** |
| --- |
| **Fig. S4** Electrografting of primary aliphatic amines via cyclic voltammetry. As the surface of GC electrodes is functionalized with amine molecules, we observed a progressive decrease of anodic currents. Each voltammogram represents the average and standard deviation of 8 electrodes. Scan rate of 0.1 V/s. Electrolyte: 10 mM of aminohexane and 0.1 M of TBATFB prepared in acetonitrile. |

| **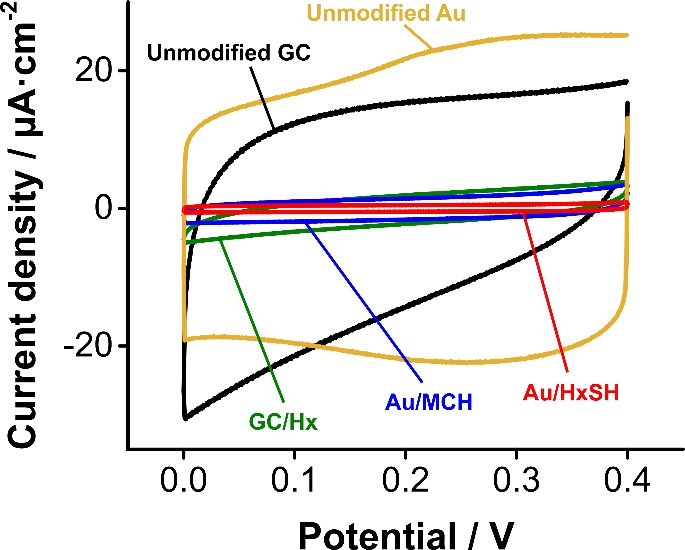** |
| --- |
| **Fig. S5** Cyclic voltammograms of alkanethiol-based and amine-based monolayers on gold and carbon electrodes, respectively. We show current densities to account for differences in electrode areas. Doing this allows us to directly compare voltammetric charging currents for the different systems studied. Voltammograms taken in PBS at 0.1 V/s. Each trace represents the average of 4 electrodes. |

| **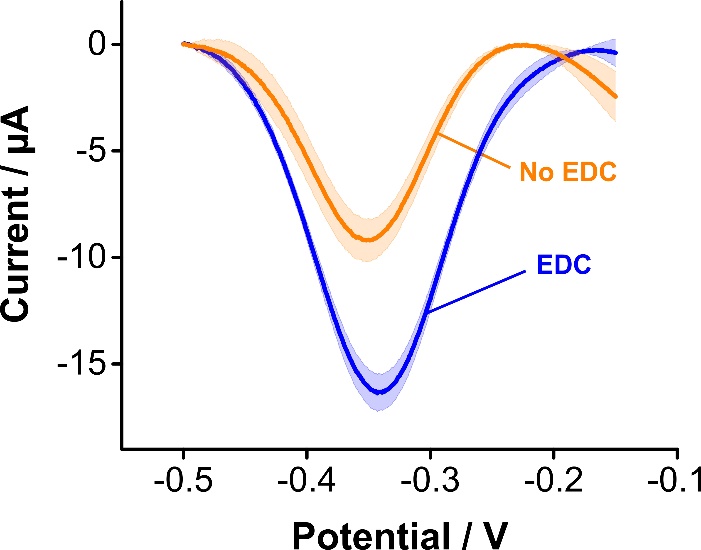** |
| --- |
| **Fig. S6** EDC/NHS coupling of amine-modified tobramycin-binding aptamer to electrografted aminohexanoic monolayers on carbon electrodes. We compare control measurements for functionalization procedures without EDC/NHS (yellow trace) and with EDC/NHS (blue trace). SWVs taken in PBS using a frequency of 200 Hz, an amplitude of 50 mV, and a step size of 1 mV. Each trace represents the average of 4 electrodes. |

| **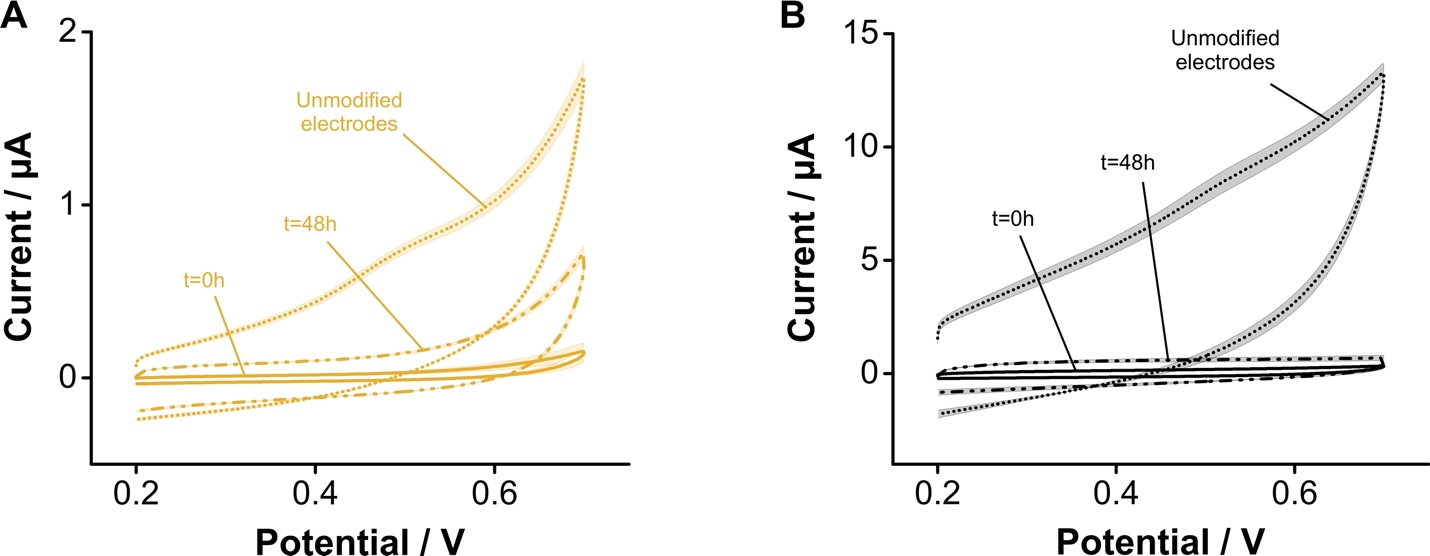** |
| --- |
| **Fig. S7** Change of cyclic voltammograms in undiluted serum after 48 hours of continuous voltammetric interrogation and comparison of the response obtained with unmodified (**A**) gold, and (**B**) glassy carbon electrodes. Each voltammogram represents the average and standard deviation of 4 electrodes. Voltammograms taken at 0.1 V/s. |
